# A systematic review of alterations in brain activation and intensity following stroke: implications for integration and functional outcomes

**DOI:** 10.3389/fneur.2025.1544008

**Published:** 2025-05-26

**Authors:** Amirul Azri, Noorazrul Yahya, Umi Nabilah Ismail, Muhammad Aminuddin Ashari, Hanani Abdul Manan

**Affiliations:** ^1^Makmal Pemprosesan Imej Kefungsian (Functional Image Processing Laboratory), Department of Radiology, Universiti Kebangsaan Malaysia, Kuala Lumpur, Malaysia; ^2^Diagnostic Imaging and Radiotherapy Program, Centre of of Diagnostic and Applied Health Sciences, Faculty of Health Sciences, Universiti Kebangsaan Malaysia, Kuala Lumpur, Malaysia; ^3^Department of Radiology, Hospital Canselor Tuanku Muhriz, Universiti Kebangsaan Malaysia, Kuala Lumpur, Malaysia; ^4^Department of Radiology and Intervency, Hospital Pakar Kanak-Kanak (Children Specialist Hospital), Universiti Kebangsaan Malaysia, Kuala Lumpur, Malaysia

**Keywords:** ischemic stroke, hemorrhage stroke, based functional magnetic resonance imaging, rehabilitation, brain activity, neurorehabilitation, compensation

## Abstract

**Background:**

Stroke remains a leading cause of disability, making it critical to understand the underlying neurophysiological mechanisms for effective rehabilitation. Task-based functional Magnetic Resonance Imaging (fMRI) provides valuable insights into brain activation patterns; however, its correlation with clinical evaluations is not yet fully understood. This systematic review aims to explore the relationship between task-based fMRI findings and clinical assessments in stroke patients, comparing them to healthy controls.

**Methods:**

Using the PubMed database and adhering to PRISMA guidelines, we identified and analyzed 11 eligible studies. Total participant is 323 participant with 258 of them is stroke patients and 65 is HC.

**Results:**

Results reveal significant differences in brain activation patterns between stroke patients and healthy controls, with stroke patients displaying compensatory hyperactivation in certain brain regions such as cerebellum, inferior parietal cortex, and contralesional area. Additionally, longitudinal comparisons among stroke patients show notable improvements in activation patterns from baseline to the subacute and chronic stages following rehabilitation. These changes align with enhanced clinical outcomes, suggesting that fMRI may serve as a sensitive biomarker for recovery progress. Importantly, correlations between fMRI results and clinical scores highlight the potential of task-based fMRI to inform and refine rehabilitation strategies.

**Conclusion:**

This review underscores the value of integrating fMRI findings into clinical practice to better understand stroke recovery mechanisms. Further research is needed to deepen our understanding of these associations and optimize patient outcomes in stroke rehabilitation.

## Highlights

- Increased brain activity and activation intensity are linked to improved clinical outcomes in stroke patients.
- Neuroplasticity plays a key role in stroke recovery by enabling brain reorganization.
- Targeted rehabilitation strategies can effectively promote neuroplasticity, aiding motor and cognitive recovery.
- Genetic factors, such as variations in BNDF gene, influence recovery, with certain variants linked to poorer outcomes.
- Biomarkers, like perineal angiogenesis, can provide insights into stroke recovery trajectories and prognosis.
- The findings emphasize the importance of personalized rehabilitation approaches to optimize recovery.

## Introduction

Stroke is a condition where blood. Does not reach any part of the brain, leading to consequences such as loss of motor control, speech difficulties, and cognitive impairments. This disruption can result in various disabilities, depending on the area of the brain affected (1). Early intervention and rehabilitation are essential for improving recovery outcomes and enhancing the quality of life for stroke survivors (2). Utilizing functional magnetic resonance imaging (fMRI) to assess the specific impacts of stroke on an individual can inform targeted therapies and support systems, enabling more personalized rehabilitation approaches.

fMRI is a powerful, non-invasive tool widely used in healthcare environments. By measuring brain activity through changes in blood flow, fMRI can provide critical insights into brain functions and is valuable for research purposes, diagnosis, and treatment planning (3). Its ability to illustrate dynamic brain processes in real-time has transformed our understanding of the human brain, making it a cornerstone of modern medical imaging.

fMRI analysis can be divided into two types: resting-state or task-based. The general overview of the resting state does not include any task during the MRI process, while task-based involves specific tasks. This review only includes task-based fMRI analysis, as it provides insights into brain function during specific cognitive or sensory tasks, it allows us to observe how brain regions engage in response to specific stimuli or cognitive processes. When relating brain analysis to clinical assessment, task-based fMRI offers a powerful method for understanding a variety of neurological and psychological conditions. The integration of both task-based fMRI and clinical assessment can provide: (1) diagnosis and classification, (2) evaluation of treatment effect, (3) prediction of outcomes, and (4) research development.

Task-based fMRI offers a multifaceted approach to neurological diagnosis and treatment. By identifying abnormal brain activity patterns associated with specific disorders, fMRI aids in accurate diagnosis. For instance, changes in language task activation can assist in diagnosing aphasia (4), while altered motor task activation may indicate conditions such as Parkinson’s disease or multiple sclerosis (3, 5, 6). Furthermore, fMRI is instrumental in evaluating treatment efficiency by monitoring changes in brain activity before and after interventions. This allows clinicians to assess treatment responses and tailor therapies to individual patient needs. Additionally, fMRI can predict disease progression or recovery trajectories by analyzing initial brain activity and connectivity patterns (7, 8). This predictive capability informs clinical decision-making and provides patients with a clearer understanding of their treatment journey. Moreover, fMRI research facilitates the development of novel clinical assessment tools and therapeutic approaches by uncovering detailed brain function profiles related to specific tasks (9). By isolating specific brain regions activated during particular activities, researchers can gain a deeper understanding of cognitive processes and inform the creation of more effective diagnostic criteria and treatment modalities (10).

Integrating task-based fMRI with clinical assessments provides valuable insights into the neurophysiological mechanisms underlying stroke recovery. However, the precise correlation between brain activation patterns and clinical evaluations, particularly in comparisons between stroke patients and healthy controls, remains insufficiently understood. This gap warrants further exploration.

## Methodology

### Search strategy and selection criteria

Studies were found through a variety of methods that included computerized bibliographic searches and review of reference lists of pertinent articles according to Preferred Reporting Items for Systematic Reviews and Meta-Analyses (PRISMA) guidelines (11). The database used was PubMed (National Centre of Biotechnology) to identify the articles and searched on 23^rd^ May 2024. The keywords used were (((ischemic stroke)) OR (haemorrhage stroke)) AND (((((task-based fMRI) OR (tb-fMRI)) OR (task-based functional MRI)) OR (task-based functional magnetic resonance imaging))).

**Figure 1:**
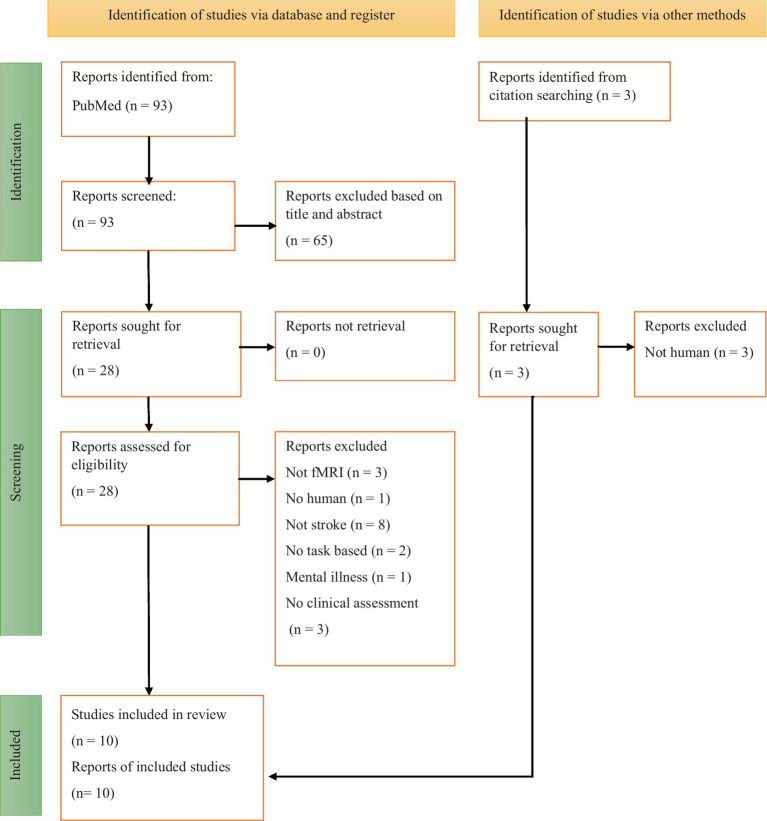
Flow diagram of the PRISMA study selection progress.

A total of 94 articles were generated from the database, as per Figure 1 (PICOS criteria for eligibility selection). These articles were transferred onto Google Sheets to aid with the screening progress. The PICOS criteria were employed to define the inclusion and exclusion parameters for article selection. Table 1 presents an outline of the criteria. The 93 articles were independently screened based on title and abstract. Studies were excluded if they did not involve humans, did not use task-based fMRI, did not analyze fMRI data, were conference abstracts, did not include stroke patients, or if the authors did not mention clinical assessments.

**Table 1:** PICOS criteria for inclusion in the systematic review.

PICOS criteria	
P - Population	Adults age above 18 years old with a stroke
I - Interventions	fMRI with task-based
C - Comparison	Healthy controls vs. stroke, stroke patients after rehabilitation, stroke with biomarkers
O - Outcome	Brain activation and intensity of activation, correlation between stroke and clinical outcomes
S - the type of study	Exclude studies with no statistical comparisons (case study or case series), reviews, editorials, conference papers, non-English, and non-human studies.

fMRI, Functional magnetic resonance imaging.

### Eligibility assessment

Twenty-eight articles were selected to undergo full-text screening, and eighteen more articles were further excluded from the review. One of the co-authors, HAM conducted a review and verification of the final ten articles. These articles underwent evaluation, and essential relevant data was summarized in a table for incorporation into the study. The search strategy, following PRISMA guidelines and the selected articles are documented in Figure 1 and following previous studies (12–19).

### Data extraction

After selecting the appropriate articles, the following details were gathered: (a) study author(s), (b) year of publication, (c) country where the study was conducted, (d) number of participants, (e) average age of participants with standard deviation, (f) types of stroke, (g) time since stroke onset, (h) senses affected, (i) task conducted/task conducted to access motor function (j) versus, (k) analysis of fMRI data (Table 2). The information that was gathered underwent thorough review and discussion. In Table 3, we extracted the clinical data from each of the final articles, along with the brain activity and connectivity information, to analyze the relationship between clinical outcomes and neural patterns and implications for rehabilitation.

**Table 2:** Demographic table of reviewed article.

Authors (Years), Country	No. of participants	Mean age SD (range)	Comparison	Type of stroke	Post-stroke onset	Assessed functional areas	Task/Paradigm	Brain infarct location
Du et al. (2018) (22), China	34 (8F 26M)	52	Stroke only (baseline and after 3 months)	Ischemic	2 weeks	Motor	Finger tapping	MCA, SMA
Bajaj et al. (2015) (2), USA	10 (4F 6M)	60.1 ± 10.52 years	Stroke only	Ischemic and hemorrhage		Upper limb motor	Motor imaginary task, motor execution task	M1, PMC, SMA
Kreydin et al. (2020) (1), California	17	(18-60stroke)(18–40 HC)	Stroke and healthy	Cerebral stroke	15–33 months	Lower urinary tract	Urodynamic cycle	Cerebellum, periaqueductal grey, bilateral insula
Kristinsson et al. (2019) (10), Columbia	87	(21–80)	Stroke only (BDNFgenotype)	Ischemic	9.5 months	Aphasia	Picture naming task	MCA, posterior occipital and parietal lobe, anterior temporal and frontal area
Nardo et al. (2017) (4), UK	18 (6F 12M)	50 ± 12	Stroke only (baseline and after 6-weeks)	Ischemic	61 months ± 58	Aphasia	Picture naming task	L hemisphere
Saleh et al. (2011) (21), USA	4 (2F 2M)	61.5 ± 7 years (55–70)	Stroke only (baseline and after 8 days)	Ischemic	have value but no parameter	Sensorimotor, motor	Full hand flexion	Cerebrovascular
Bonakdarpour, Parrish, and Thompson (2007) (20), USA	5 (1F 4M)	(36–65)	Stroke and healthy	Ischemic and hemorrhage	2 years	Aphasia	Words and pseudowords	Broca area, posterior perisylvian network
Meehan et al. (2011) (25), Canada	18 (8F 10M)	(55–74)	Stroke and healthy	Ischemic	12 months	Motor	Joystick based task	PMC
Li et al. (2021) (23), China	21 (12F 9M)	21 ± 9	Stroke only	Ischemic		Sensorimotor, motor	Finger to thumb task	SM1, PA
Wang et al. (2022) (24), China	105 (40F 65M)	(59.56 ± 9.82-stroke) (56.64 ± 5.17)	Stroke and healthy	Ischemic	27.22 ± 17.53 (Days)	Sensorimotor, motor	Thumb- index finger tapping	Ipsilesional hemisphere

MCA, middle cerebral artery; SMA, supplementary motor area; M1, primary motor cortex; PMC, premotor cortex; SM1, primary sensorimotor cortex; PA, perinidal angiogenesis; M, male; F, female.

**Table 3:** Clinical assessment and brain activity of reviewed article.

Authors (Years)	Clinical assessment	Brain connectivity and correlation	Brain area activation
Du et al. (2018) (22)	Motor deficit and stroke disability assessments show improvement after 3 months from baseline		Affected hand: enhanced activity in ipsilesional M1, PMC, SMA and contralesional hemisphere after 3 months post-baseline
Bajaj et al. (2015) (2)	FMA shows improvement after training.	*Motor imagery* Affected hand—increase significant bidirectional connections (SMA<->PMC, PMC<->M1) after training, whereas only one connection (PMC -> M1) at baseline. Unaffected hand—increase significant bidirectional connection (PMC<->M1, between SMA<->M1) after training, whereas only one connection (M1 -> PMC) at baseline. *Motor execution* Affected hand—increase significant connection after training (SMA<->PMC) compared to baseline (SMA -> M1) Unaffected hand—increase of significant connection (PMC<->M1, SMA<->M1) compared to baseline (M1<->PMC)	
Kreydin et al. (2020) (1)	HC has less severity of overactive bladder compared to stroke patients HC has lower level of distress overactive bladder compared to stroke patients		*Cerebellum*—activation in Stroke > HC. *Motor cortex & SMA*—activation in HC > Stroke. *Cingulate Cortex (anterior & posterior)*—HC > stroke patient. *Dorsolateral Prefrontal Cortex*—activation only in HC during *void vs. rest* contrast *Insula & Periquaductal Grey*—activation only in stroke *Inferior Parietal Cortex*—activation only in stroke
Kristinsson et al. (2019) (10)	Typical BDNF have lower severity of aphasia compared to atypical BDNF Typical BDNF have higher accuracy of semantic memory and language abilities compared to atypical BDNF		*Activation pattern*—Both genotypes shows similar patterns of activation focusing on bilateral posterior temporal gyrus, pre- and postcentral gyrus and longitudinal fissure *Activation intensity*—typical BDNF shows higher intensity of activation compared to atypical BDNF (R_posterior temporal lobe, R pre-and postcentral gyrus and R frontal lobe white matter)
Nardo et al. (2017) (4)	Stroke patients have higher accuracy after 6 weeks training compared to the baseline Stroke patients have a higher reaction time after 6 weeks training compared to baseline There was a significant positive effect of anomia training on the naming performance improving accuracy and reaction time		*R_anterior insula & R_IFC*- increase in BOLD response after training compared to baseline. *R_dACC & L_PMC* –increase of BOLD response after training compared to baseline.
Saleh et al. (2011) (21)	Movement duration was shorter after 8 days training compared to baseline increase of angular excursion after 8 days of training compared to baseline increase in angular velocity after 8 days of training compared to baseline	*Ipsilesional motor cortex & bilateral sensorimotor area* –increase connectivity after training compared to baseline. Patients with subcortical lesions shows smaller increase *Contralesional hemisphere* –Active voxel in Subject 1: post-training<baseline *Ipsilesional hemisphere*—Active voxel in Subject 3 and 4: post-training>baseline.	Subjects 1 and 2 showed more activation in the motor cortex and sensorimotor area, while Subjects 3 and 4 showed less activation compared to baseline.
Bonakdarpour et al. (2007) (20)	HC have higher accuracy compared to stroke patients HC have lower reaction time compared to stroke patients		*Perisylvian Region*—HC and A2 & A5 patients have lower BOLD TTP compared to other stroke patients. *Visual Cortex*—HC and stroke patients shows no different in BOLD TTP in Left primary visual cortex and Right occipital area.
Meehan et al. (2011) (25)	HC have less tracking error on the baseline (day 1 training) compared to stroke patients HC have lower tracking error compared to stroke patients after 6 days training even after improvement in both groups.		*Dorsolateral Prefrontal Cortex* Random sequence tracking - HC shows positive BOLD response while stroke patient shows no difference in BOLD response after 6 days training Repeated sequence tracking—HC shows negative BOLD response compared to stroke patients with no difference in BOLD response. After 6 days training *PMd* Random sequence tracking—HC and stroke patients shows smaller positive BOLD response Repeated sequence tracking—HC shows higher positive BOLD response compared to BOLD response in stroke patients
Li et al. (2021) (23)	Presence of PA-HK distance was significantly associated with lower interhemispheric activation ratio there no correlation was found between nidus volume and interhemispheric activation ratio		*Contralesional hemisphere*—All stroke patients have activation center located within hand knob area SM1 *Ipsilesional Hemisphere*—most patients (17 out of 21) activation center are within the hand knob area. One patient shifted activation center laterally from the hand knob area. Three patients have no significant activation detected
Wang et al. (2022) (24)	Patients with nearly no functional damage have less increase and decrease connections within the contralesional hemisphere and between bilateral hemisphere. patients with mild functional damage (NIHSS 2–4) had the strongest activations within contralesional hemisphere and between bilateral hemisphere	*Ipsilesional hemisphere*—HC have lower positive functional correlations between precentral and postcentral gyri compared to stroke patients. *Contralesional hemisphere*—HC and stroke patients have similar lower value positive correlations between precentral and postcentral gyri *Bilaterael upper limb motor regions*—HC have smaller activation range compared to stroke patients in ISC. *Interhemisphere correlations*—HC and stroke patients have same number of positive and negative correlations between ROI.	

MCA, middle cerebral artery; SMA, supplementary motor area; M1, primary motor cortex; PMC, premotor cortex; PMd, dorsal premotor cortex; SM1, primary sensorimotor cortex; PA, perinidal angiogenesis; FMA, Fugl-Meyer Assessment; ROI, region of interest; HC, healthy control; ISC, inter-subject correlation; BOLD, blood oxygenation level dependent; TTP, time to peak; R_, right; L_, left; dACC, dorsal anterior cingulate cortex; IFC: Inferior frontal cortex.

## Results

### Study characteristics

The literature search produced 94 articles; however, only 10 relevant articles met the PICOS criteria for the systematic review. The selected articles were published between 2007 and 2022 and covered a range of stroke-affected functional areas and task-based fMRI findings. The majority of the studies included in the review were conducted in the USA (1, 2, 20, 21), comprising a total of four studies. Additionally, three studies originated in China (22–24), while there was one study each from Colombia (10), Canada (25), and the UK (4). The total number of participants in this review is 357 participants, 160 were male and 93 were female. Two articles did not report the gender of the subjects (1, 10). Participants included in the studies had an average age range of 18 to 80 years.

The majority of articles (*n* = 7) focus exclusively on ischemic stroke (4, 10, 21–25), while two articles address both ischemic and hemorrhagic stroke (2, 20), and one article specifically examines cerebral stroke (1). The reported onset of stroke varied widely, with duration ranging from subacute (2 weeks) to chronic (up to 5 years), although three studies did not specify participants’ stroke on set (2, 21, 23).

The predominant focus of the articles is on the motor area and sensorimotor area, with seven studies addressing these topics (1, 2, 21–25). In contrast three articles center on the speech-language area (4, 10, 20). Each article employed various task paradigms specifically designed to meet its objectives. For instance, studies focused on the speech-language area often utilized tasks such as picture naming (4), while those investigating the motor area typically included tasks like finger tapping or full hand flexion (21, 22). Details of the tasks conducted for each article can be seen in Table 2.

The studies included in the review can be categorized into two distinct types. The first category compared various aspects of stroke among stroke patients (six studies) (2, 4, 10, 21–23). The study includes; analyzing changes observed before and after training (4, 21, 22). One study reported the differential impact of genetic backgrounds on post-stroke outcomes (10). One study reports on how stroke affects cognitive and motor functions differently using resting state and task-based conditions (23). Finally, one study reports the best fit dynamic causal modeling among stroke patients (2).

The second category compared the differences between stroke patients and healthy controls (1, 20, 24, 25). This comparative approach is instrumental in discerning variations in brain function, cognitive abilities, and functional connectivity between individuals who have experienced a stroke and those who have not.

### Effect of functional disorders post-stroke on brain activation

#### Motor function

Motor impairment is a neurological condition that affects an individual’s ability to move, coordinate, and control muscle movements. Motor dysfunction can significantly affect daily activities, mobility, and quality of life. This review examines 6 studies on motor dysfunction and its clinical assessment, providing insights to improve understanding, interventions, and patient outcomes.

All six articles report on the same area of the brain which are primary motor cortex (M1), premotor cortex (PMC), and supplementary motor area (SMA). Du et al. (22) reported an enhancement of brain activity in the ipsilesional M1, PMC, SMA, and contralesional hemisphere in the affected hand. After the stroke patients went through a rehabilitation process for 3 months, and all patients had improvement in motor deficit and stroke disability assessment.

Bajaj et al. (2) correlated brain activity with the Fugl-Meyer Assessment (FMA) score. The author found that stroke patients showed an improvement after training compared to baseline. During motor imagery tasks, the affected hand shows significant bidirectional connections between the SMA and the PMC and between PMC and M1. During motor execution tasks, the affected hand shows a lower significant connection between SMA and PMC after training. During both motor imagery and motor execution tasks, unaffected hands showed a significant bidirectional connection between PMC and M1, and between SMA and M1 compared to baseline whereby only connection from M1 to PMC was found.

Saleh et al. (21) used virtual training on stroke patients to demonstrate the effect of virtual training on motor dysfunction. The author reported an increase in angular excursion and angular velocity after 8 days of training. fMRI analysis illustrated increased connectivity after training compared to baseline in the ipsilesional motor cortex and bilateral sensorimotor areas. However, patients with subcortical lesions show smaller increments of connectivity in the same area. In the contralesional hemisphere, only one patient showed active voxels in the post-training phase compared to baseline, while others showed no active voxel. Ipsilesional hemisphere, on the other hand, shows active voxel in two patients (Patient 3 and Patient 4) in post-training compared to baseline.

Li et al. (23) investigated task-based fMRI on the hand knob area (HK) and perinidal angiogenesis (PA). The presence of PA-HK was significantly associated with lower interhemispheric activation, and there was no correlation between PA-HK nidus volume and interhemispheric activation. The authors reported all patients had an activation center located in the hand knob area within contralesional M1. For the ipsilesional hemisphere, most patients (17 out of 21) activation center are within the M1. One patient shifted the activation center laterally from HK, and three patients had no significant activation detected. These findings suggest that PA may disrupt motor activation patterns in stroke patients.

When comparing stroke patients with healthy control (HC), there are clear differences in clinical outcomes and brain activities as described in Meehan et al. (25). All participants engaged in continuous tracking of a target moving in a sine-cosine waveform by manipulating a joystick. The waveform consists of either a repeated sequence pattern or a random sequence pattern within a period. At baseline, HC showed less tracking error compared to stroke patients. After 6 days of training, stroke patients displayed gains in tracking with a smaller number of inaccuracies. fMRI analysis in random sequence tracking, HC exhibits a blood oxygenation level dependent (BOLD) response in the dorsolateral prefrontal cortex, whereas stroke patients show no response in this region. For repeated sequence tracking, in the dorsal premotor cortex (PMd), both HC and stroke patients show a small positive BOLD response during random sequence tracking. However, for repeated sequence tracking, HC has a higher positive BOLD response compared to stroke patients.

Stroke and HC exhibited high-level positive correlations between regions of interest (ROI) which were hand-related sensory-motor and imagination cortices (24). In the study, the evaluation focused on the common activation and suppression finger-tapping task. The article also states that stroke patients have a higher positive correlation among the ROI compared to HC in the ipsilesional hemisphere. In this article, the author uses intersubject correlation (ISC) and intersubject functional correlation (ISFC). Stroke patients have a wider activation range compared to HC, activating the bilateral upper-limb motor region. They also have more significant pairs within the contralateral side and more positive pairs between the bilateral and lower body ROI. The increased positive correlations in ipsilesional hemisphere indicate an attempt to strengthen connections related to motor control.

#### Language function

Three articles specifically focus on stroke patients with language impairment. Kristinsson et al. (10) observed genotype-specific differences in brain-derived neurotrophic factor (BDNF) in cortical activation patterns among stroke patients. The article suggests that typical BDNF is linked to lower aphasia severity and higher accuracy in semantic memory and language abilities compared to atypical BDNF. MRI data showed similar activation patterns in stroke patients, with the highest intensity in the posterior temporal gyrus, pre- and postcentral gyrus, and longitudinal fissure. The study suggests that typical BDNF may enhance neural activity related to language processing and semantic memory, contributing to better outcomes in aphasia patients and enhancing neuroplasticity and recovery after stroke.

Nardo et al. (4) examined the effects of anomia training in stroke patients and used the same picture during scanning. After six weeks of training, patients showed improved accuracy and faster reaction times compared to their initial performance. Brain imaging BOLD responses revealed increased activity in the right anterior insula and right inferior frontal cortex after training. Additionally, the authors noted positive changes in BOLD responses in the right dorsal anterior cingulate cortex, and left premotor cortex when comparing the results after training to the baseline measurements. The increased BOLD responses in the key regions associated with language and executive function indicate that the training may facilitate neural reorganization.

One article in this review compared HC with stroke patients in relation to language impairment. Bonakdarpour et al. (20) identified differences in hemodynamic response functions among aphasic patients when engaged with words and pseudo-words. In clinical comparisons between HC and stroke patients, HC showed higher accuracy compared to stroke patients. In terms of reaction time, HC had a lower reaction time compared to stroke patients. The author also reported on the perisylvian region and visual cortex. In the perisylvian region HC, along with two stroke patients (A2 & A7) showed lower BOLD time to peak (TTP) compared to other stroke patients. In the visual cortex, HC and stroke patients show no difference in BOLD TTP in the left primary visual cortex and right occipital area.

#### Micturition dysfunction

Kreydin et al. (1) demonstrated micturition-related brain activity in stroke patients who developed lower urinary tract (LUT) symptoms. The study reported that HC experienced less severity and lower distress related to an overactive bladder compared to stroke patients. HC showed higher brain activation than stroke patients in the PMC, SMA, and cingulate cortex (anterior and posterior). Stroke patients have higher activation than HC in the cerebellum. Dorsolateral prefrontal cortex activation occurred only in HC. However, stroke patients have activation in the insula, periaqueductal grey, and inferior parietal cortex. These findings suggest that stoke patients with LUT symptoms may experience Altered neural responses during micturition, reflecting the complex interplay between motor control and bladder functions. The activation of insula and periaqueductal grey in stroke patients highlights their role in the emotional and autonomic aspects of bladder control.

### Task-based fMRI paradigms

All the reviewed articles use a block diagram, which consists of rest and stimulation alternating periods. The alternating rest and stimulation phases help distinguish between baseline brain activity and activity induced by specific tasks as in Table 4. In this review, we found three main observed functional areas, motor, language and urinary tract. We have six articles examining motor function (2, 21–25), three examining speech language function (4, 10, 20) and one article examining urinary track (1). Three articles use finger taping, and one article each for motor imaginary, hand flexion and joystick tasks. Finger tapping and hand flexion challenge fine motor coordination and speed, engaging areas like M1, PMC, and SMA. Motor imagery has been shown to activate similar brain regions as actual movement, including the M1 and prefrontal cortex. In the joystick task, participants are given a sine cosine wave, and they need to follow the wave using the joystick movement. Joystick tasks involve the coordination of hand movement with dynamic visual feedback, activating M1 as well parietal cortex. In language impairment articles, authors use picture naming with two article reports and words and pseudowords with one report. Picture naming involves presenting participants with pictures of objects and asking them to name them aloud. This is linked to activation in Broca’s area, Wernicke’s area, and angular gyrus. Words and pseudowords recognition test the brain’s ability to recognize and process familiar words forms. Word recognition typically engages areas in the left posterior lobe and angular gyrus. One article uses urodynamic cycle to observe brain activation. Participants asked to undergo simulation of the filling, and voiding. This task aims to understand how the brain processes sensation of bladder fullness and urgency. All these paradigms are designed to investigate how different types of stimuli alter brain activity and provide insight into neural mechanisms involved in each functional area.

**Table 4:** Motor impairment and task paradigms of the reviewed articles.

Authors (year)	Affected functional area	Task paradigm
Du et al. (2018) (22)	Motor	Finger tapping
Bajaj et al. (2015) (2)		Motor imaginary and execution task
Saleh et al. (2011) (21)		Full hand flexion
Meehan et al. (2011) (25)		Joystick task
Li et al. (2021) (23)		Finger tapping
Wang et al. (2022) (24)		Finger tapping
Kristinsson et al. (2019) (10)	Language	Picture naming
Nardo et al. (2017) (4)		Picture naming
Bonakdarpour et al. (2007) (20)		Words and pseudowords
Kreydin et al. (2020) (1)	Urinary tract	Urodynamic cycle

### Limitation of included studies

This review uncovers limitations shaped by the authors’ experiences. Key challenges include small sample sizes and striking differences in group sizes, which could impact the findings. Additionally, the authors report technical limitations, such as structural image flipping from left to right (22). They also mention pre-experiment procedural issues, such as not measuring blood flow before the MRI scan, which affects the hemodynamic responses of patients (20), as well as diverse stroke latency in patients (2) and the absence of urodynamic evaluation (1). Furthermore, the articles highlight uncontrolled external factors that could slightly affect the results, such as the heterogeneity of rehabilitation among patients (22), individual patient behavior and brain deficits (2, 20), and motivation or social support received by the patients (10). The authors also report a deficiency of data, such as the exclusion of perfusion imaging data, which could enhance the understanding of blood flow (23).

Additionally, the authors express dissatisfaction with their findings for several reasons: focusing on only one set of findings, which limits the ability to correlate results (10); the etiology of stroke, which includes both hemorrhagic and ischemic types (20); the possibility that the results are due to compensation by other regions (23); and differences in movement kinetics among patients (21).

## Discussion

In this systematic review, we aim to examine alterations in brain activity and intensity of activation in stroke patients, and how these changes correlate with clinical outcomes. The review synthesizes findings from various reports that detail how modifications in brain activity and activation intensity relate to clinical progress. Generally, improvements in clinical outcomes are associated with positive changes in brain activity and activation intensity, regardless of whether the training session spans a few days or several months. This is evident in articles focusing on the motor areas of the brain, which report greater activation in the SMA and PMC (2, 22), while studies examining language and speech highlight activation in Broca’s area and the perisylvian region (4, 20).

### Brain compensation mechanisms post-injury

The brain’s ability to compensate after injury is a remarkable example of its plasticity, or its capacity to reorganize and adapt to new circumstances. Following an injury such as stroke, the brain often undergoes significant changes in response to the loss of function in certain areas. This process of functional compensation allows other parts of the brain to take over the responsibilities of the damaged regions.

Neuroplasticity is one of the fundamental aspects of post-injury brain compensation, which is the brain’s ability to form a new neural connection. Neuroplasticity allows for the recovery of lost functions, but it can also lead to the recruitment of alternative regions that were not originally involved in the lost function. Same as Wang et al. (24) which reported an increase in activation in motor areas of the opposite hemisphere from the affected hemisphere. Initially, the ipsilesional hemisphere often exhibits reduced activity due to the damage caused by the stroke, which impairs motor function on the affected side. However, as recovery progresses, excitability in the opposite hemisphere gradually increases influenced by neuroplasticity and rehabilitation efforts. This supports previous findings indicating that ipsilesional corticomotor excitability, initially suppressed leads to an increase over time while activation in unaffected hemisphere rises (26). Meanwhile, the contralesional hemisphere often compensates by becoming more active, which can support recovery but may also lead to maladaptive changes if overly dominant (27, 28).

Compensatory mechanisms have significant implications for rehabilitation. By understanding how the brain reorganizes itself after injury, rehabilitation strategies can be designed to enhance these compensatory processes.

### Task-based fMRI paradigms on promoting neuroplasticity

Task-based fMRI paradigms play a key role in promoting neuroplasticity by engaging the brain in structured, repetitive activities that target specific motor or cognitive functions. Tasks such as hand flexion or joystick control challenge the brain to reorganize its neural circuits, particularly after neurological injuries like stroke (21, 25). The more these tasks are performed, the more likely new neural pathways will form, compensating for damaged areas and aiding recovery.

Central to this process is “experience-dependent plasticity,” where repeated practice strengthens synapses and forms new neural connections. Variations in task intensity, duration, and movement type can influence brain regions activated, further promoting neuroplastic changes (53, 54). For example, language exercises can stimulate speech-related areas, leading to improved communication abilities (4). Research indicates that varying task intensity enhances language and speech recovery, as seen in studies on anomia treatment (51, 52, 56).

In clinical practice, task-based paradigms support recovery by encouraging the brain to adapt and reorganize. For stroke survivors, adjusting task difficulty and repetition promotes functional recovery, leading to greater independence in daily life (55, 57).

### Gene-related factor on stroke recovery

Recovery from a stroke is a complex, multifaceted process that involves a dynamic interaction of various factors. While much of stroke recovery focuses on rehabilitation strategies and medical treatments, recent research suggests that genetic factors may also play a significant role in shaping recovery outcomes (48). Gene influence many biological processes that are crucial for recovery, such as neuroplasticity, inflammation, cell survival, and tissue repair. Additionally, genetic variations can affect how an individual responds to different rehabilitation therapies or medications, leading to more personalized treatment approaches.

In a discussion of neuroplasticity and cognitive recovery after stroke, brain-derived neurotrophic factor (BDNF) emerges as a critical molecular player. BDNF supports synaptic plasticity, neural survival, and growth, with its secretion influenced by neuronal activity (29–31). A common genetic variation In the BDNF gene is the Val66Met polymorphism with variants of Met alleles at the polymorphic site. This variant of met alleles which linked to a poorer cognitive outcome. Previous studies have shown that the presence of met alleles results in reduced memory, learning, and also high severity of aphasia (10, 32, 33). In our review, the met alleles patient shows low accuracy of semantic and language abilities compared to non-carriers (10). To support this statement, met alleles also demonstrate poor brain activation and smaller brain activation volume which results in worse functional recovery after stroke (34–37). This indicates that genetic variations in BDNF may significantly impact recovery trajectories and underscore the importance of personalizes approaches in stroke rehabilitation.

### Training interventions: enhancing cognitive function post-stroke

Training and interventions aimed at enhancing cognitive function post-stroke have shown significant improvement in brain and clinical outcomes. Studies indicate that targeted training leads to increased brain activation and better functional connectivity, which contribute to improved daily life management (2, 21, 22, 25). These findings suggest that interventions focused on motor and cognitive functions can positively impact clinical assessment and support recovery by stimulating neural pathways involved in both motor control and cognition.

The process of neuroplasticity, whereby the brain reorganizes and forms new neural connections, plays a key role in these improvements (38). By engaging in structural rehabilitation, stroke patients can enhance brain activity and promote functional recovery. The evidence shows that targeted therapeutic approaches effectively stimulate the brain’s neural networks, enabling recovery in both motor and cognitive functions by fostering the reorganization of neural circuits.

In aphasia, a language disorder caused by stroke targeted training interventions can enhance neural activity in language-related brain areas, improving language recovery. Aphasia impacts communication, significantly affecting daily life and equality of life. Studies have shown that language-focused rehabilitation leads to increased brain activation in key areas such as Broca’s and Wernicke’s area, which supports language function recovery (4). Neuroplasticity plays a critical role in aphasia treatment, where stimulating neural pathways involved in language processing facilitates functional reorganization and communication recovery.

### Key indicators of brain function in stroke recovery

The observed finding also identifies Perinidal Angiogenesis as a predictor of brain activity (23). The author does not mention about correlation of clinical assessment with brain activity. However, the author correlates the Perinidal Angiogenesis and Hand Knob (PA-HK) presence to make the interhemispheric activation lower compared to patients that do not have PA-HK. This theory supported by the “sump effect” of arteriovenous malformations (AVMs) causes the arteriovenous to dilate and absorb the blood from nearby vessels into the nidus, which indirectly supplies it and leads to the dilation of secondary vessels (39, 40). Seconds theory is that high-flow shunts in AVMs can cause the brain to lose oxygen because of reduced blood flow and increased pressure in the veins (36, 41, 42). That concludes that PA-KH presence makes the interhemispheric activity decrease.

### Neurocognitive differences between healthy individuals and stroke patients

Understanding brain activity in normal individuals compared with stroke patients reveals significant differences that are critical for both diagnosis and rehabilitation. Despite having major differences in clinical assessments, there are variations in the context of brain activity. Research by (20) indicates that HC demonstrates higher accuracy and faster reaction times compared to stroke patients. Brain activity in HC shows lower BOLD TTP compared to stroke patients, particularly in the perisylvian regions. These regions play a key role in the encoding and decoding of speech sounds, syntactic structure, and semantic meaning (50). Furthermore, patients also demonstrate an unusual BOLD response curve, marked by extended dips and a significantly delayed positive peak. These patterns suggest that oxidative metabolism is occurring without the typical increase in cerebral blood flow (43, 44), which reduces blood flow to the brain area, thereby lowering the TTP of the BOLD signal. The observed differences in BOLD TTP between HC and stroke patients may reflect the impact of stroke-related injury on the efficiency of neural processing within these language areas. Importantly, this dysfunction highlights the potential for targeted rehabilitation strategies (49).

Along with this finding, one article by Kreydin et al. (1) Compares HC and stroke patients when encountering micturition. The results found that stroke patients exhibit more intense activity in the insula area. The insula is reported to be activated during urinary storage (45, 46). This has also been confirmed by Griffiths et al. (47). Kreydin et al. (1) hypothesize that increased activation in the insula may increase the sensation of urgency in stroke patients and create negative feedback from other regions. Reflecting on the current review, stroke patients have problems with controlling the lower urinary tract system.

### Limitation and conclusion

However, several limitations have arisen and that need to be acknowledged. The diversity of methodologies, recovery programs, and sample sizes can affect the generalizability of findings. Moreover, the differences in stroke types and patient demographics may influence the specific impacts of changes in brain activity on outcomes. Future research should aim to standardize assessment techniques and explore longitudinal outcomes to better understand these relationships.

Comparative analyses between healthy individuals and stroke patients reveal distinct patterns of brain activity, underscoring the necessity for personalized rehabilitation approaches. These findings advocate for further exploration of the mechanisms of neuroplasticity, with the ultimate goal of optimizing stroke recovery strategies and improving the quality of life for stroke survivors.

This systematic review highlights the crucial relationship between changes in brain activity and clinical outcomes in stroke patients. The findings consistently demonstrate that increased brain activity and activation intensity are associated with improved functional outcomes, emphasizing the effectiveness of targeted rehabilitation strategies. Neuroplasticity emerges as a key mechanism, facilitating the reorganization of neural pathways essential for motor and recovery of motor and cognitive functions. Targeted interventions, particularly in patients with aphasia, not only enhance motor and language abilities but also promote overall recovery. Additionally, biomarkers such as BDNF and perinidal angiogenesis provide valuable insights into patients’ prognoses and recovery trajectories.

## Data Availability

The original contributions presented in the study are included in the article/supplementary material, further inquiries can be directed to the corresponding author.
